# I prostanoid receptor activation attenuates pressure overload-induced cardiac hypertrophy by enhancing glucose oxidation

**DOI:** 10.1038/s41392-023-01541-1

**Published:** 2023-09-06

**Authors:** Qian Liu, Guizhu Liu, Yujuan Zhuo, Shihong Chen, Yinghong Zheng, Kai Zhang, Song Xiang, Jiangping Song, Liming Yang, Ying Yu

**Affiliations:** 1 Department of Pharmacology, Tianjin Key Laboratory of Inflammatory Biology, Center for Cardiovascular Diseases, Haihe Laboratory of Cell Ecosystem, Key Laboratory of Immune Microenvironment and Disease (Ministry of Education), The Province and Ministry Co-sponsored Collaborative Innovation Center for Medical Epigenetics, School of Basic Medical Sciences, Tianjin Medical University, Tianjin, China; 2 School of Medicine, Jiangnan University, Wuxi, Jiangsu China; 3 Department of Pathophysiology, Key Laboratory of Cardiovascular Pathophysiology, Harbin Medical University, Harbin, Heilongjiang China; 4 Department of Biochemistry and Molecular Biology, School of Basic Medical Sciences, Tianjin Medical University, Tianjin, China; 5 State Key Laboratory of Cardiovascular Disease, Fuwai Hospital, National Center for Cardiovascular Diseases, Chinese Academy of Medical Sciences and Peking Union Medical College, Beijing, China; 6 Department of Pathophysiology, School of Basic Medical Sciences, Harbin Medical University-Daqing, Daqing, Heilongjiang China; 7 State Key Laboratory of Frigid Zone Cardiovascular Diseases (SKLFZCD), Harbin Medical University, Harbin, Heilongjiang China

**Keywords:** Cardiology, Cardiovascular diseases

**Dear Editor**,

Heart failure (HF) is one of the leading causes of mortality and morbidity worldwide. Despite current treatments can improve cardiac dysfunction in HF patients, the overall mortality rate remains high, indicating more effective therapeutic strategies for HF are needed. The pathogenesis of HF can be placed into ischemic cardiomyopathy and non-ischemic cardiomyopathy, such as pressure-oveload. Unlike ischemia-induced infarctions, prolonged pressure overload leads to cardiac hypertrophy or dilated cardiomyopathy.^1^ Although the pathology of heart failure being multifactorial, it is generally accepted that the failing heart is cardiac energy deficiency or metabolic dysfunction. A metabolic switch from mitochondrial oxidation to glycolysis contributes to the development of cardiac hypertrophy and HF.^2^ Therefore, targeting energy metabolism may be an effective therapeutic approach to improving cardiac function in the failing heart. Prostaglandin (PG) I_2_ is a bioactive lipid mediator derived from arachidonic acid through sequential catalysis by two cellular bioenzymes: cyclooxygenases and PGI_2_ synthase. It is also a potent vasodilator and endogenous inhibitor of platelet aggregation and displays pleiotropic cardio-protective effects through I prostanoid receptor (IP).^3^ The PGI_2_/IP axis promotes hepatic gluconeogenesis in response to fasting in mice.^4^ However, whether and how the PGI_2_/IP axis regulates energy metabolism in the failing heart remains to be determined.

Here, we found IP inhibitor Cay10441 markedly suppressed basal respiration and maximal respiratory capacity in neonatal rat ventricular myocytes (NRVMs) among PG antagonists (Supplementary Fig. S1a, b), without a significant influence on glycolysis or glycolytic capacity (Supplementary Fig. S1c, d). IP agonist Cicaprost increased basal respiration and maximal respiratory capacity in cardiomyocytes (Supplementary Fig. S1e–j). In neonatal mouse cardiac myocytes (NMCMs), IP deficiency also decreased basal respiration and maximal respiratory capacity (Fig. 1a). The carnitine palmitoyl transferase 1 inhibitor Etomoxir and the glutaminase inhibitor BPTES were used to block fatty acid and glutamine oxidation, respectively. Pre-treatment with Etomoxir and BPTES failed to diminish Cay10441-decressed mitochondrial oxidation in cardiomyocytes (Supplementary Fig. S1k–l), while inhibition of mitochondrial pyruvate uptake using UK5099 abolished the effects of Cay10441 (Supplementary Fig. S1m, n). Thus, IP receptor activation enhances aerobic glucose oxidation in cardiomyocytes.

**Fig. 1:**
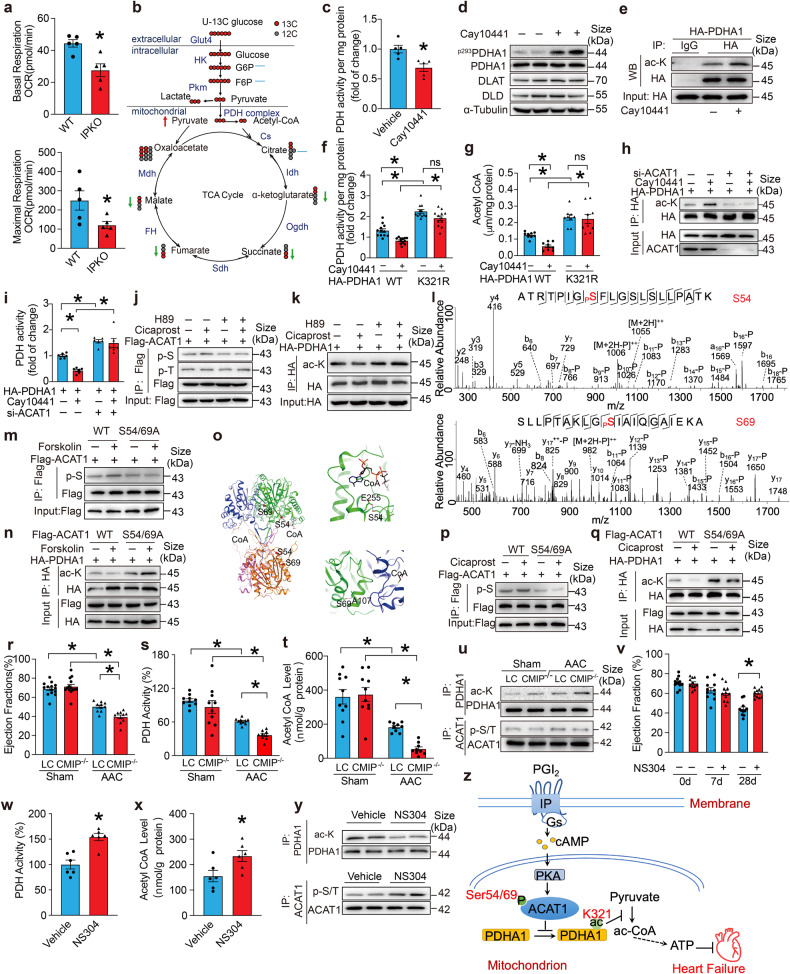
I prostanoid receptor (IP) activation attenuates pressure overload-induced heart failure by enhancing glucose oxidation. **a** Basal respiration and maximum respiration in IP KO and WT NMCMs in the presence of glucose, pyruvate and glutamine. (*n* = 5, two-sided *t* test, *P < 0.05). **b** The variation of ^13^C-glucose metabolic flux in Cay10441-treated cardiomyocytes. Fractional enrichments of glucose-6-phosphate (G6P, m + 6), pyruvate (m + 3), lactate (m + 3), and fructose-6-phosphate (F6P, m + 6), citrate (m + 1-m + 6), alpha ketoglutarate (m + 1-m + 5), succinate (m + 1-m + 4), Fumarate (m + 1-m + 4) and malate(m + 1-m + 4) as shown. Red up-arrow indicates upregulation in Cay10441-treated cardiomyocytes; green down-arrows indicate downregulation in Cay10441-treated cardiomyocytes. **c** Effect of Cay0441 treatment on PDH activity in Ang II-stimulated HL-1 cells (*n* = 5, two-sided *t* test, *P < 0.05). **d** Western blot analyses of the effect of Cay10441 on protein expression levels of PDHA1, PDHA1,^p293S^ DLAT, and DLD in Ang II-stimulated HL-1 cells. **e** Western blot analyses of the effect of Cay10441 treatment on acetylation of PDHA1 in Ang II-stimulated HL-1 cells. **f** Effect of acetyl-deficient K → R mutant of PDHA1 at 321 lysine (K321R) on PDHA1 activity in Cay10441- and Ang II-stimulated HL-1 cells. The PDH activity in untreated HL-1 cells was normalized. (*n* = 6, one-way ANOVA, Tukey multiple comparisons test was used to compare the mean of each group, *P < 0.05). **g** Effect of acetyl-deficient K → R mutant of PDHA1 at 321 lysine (K321R) on mitochondrial Acetyl-CoA level in Cay10441- and Ang II-stimulated HL-1 cells. (*n* = 9, two-way ANOVA, Tukey multiple comparisons test was used to compare the mean of each group, *P < 0.05). **h** Western blot results of the effect of ACAT1 silencing on PDHA1 acetylation in Cay10441-treated HL-1 cells. **i** Effect of ACAT1 silencing on PDH activity **i**n Cay10441-treated HL-1 cells (*n* = 6, two-way ANOVA, Tukey multiple comparisons test was used to compare the mean of each group, *P < 0.05). **j** Western blot analysis of the effect of PKA inhibitor H89 on Cicaprost-induced ACAT1 phosphorylation in Ang II-stimulated HL-1 cells. **k** Western blot analysis of the effect of PKA inhibitor H89 on Cicaprost-induced suppression of PDHA1 acetylation in Ang II-stimulated HL-1 cells. **l** MS/MS spectra showing PKA-mediated phosphorylation of ACAT1 Ser54 (top) and Ser69 (upper). The in vitro PKA kinase assay was performed using synthetic peptides ATRTPIGSFLGSLSLLPATK or SLLPTAKLGSIAIQGAIEKA, followed by LC-MS/MS analysis. **m** Western blot analysis of the effect of the ACAT1 S54/S69A double mutation on forskolin-induced ACAT1 phosphorylation in Ang II-stimulated HL-1 cells. **n** Western blot analysis of the effect of the ACAT1 S54/S69A double mutation on PDHA1 acetylation in forskolin and Ang II-stimulated HL-1 cells. **o** Structural basis of ACAT1 inactivation by Ser54 and Ser69 phosphorylation. The left panel shows the location of Ser54 and Ser69 (red spheres) in the tetrameric ACAT1 holoenzyme (PDB 2IBW). The four subunits of the enzyme are represented by different colors. The right panels show structural elements surrounding Ser54 and Ser69. Dashed lines indicate potential hydrogen bonds. **p** Western blot analysis of the effect of the ACAT1 S54/69A mutation on Cicaprost-induced ACAT1 phosphorylation in Ang II-stimulated HL-1 cells. **q** Western blot analysis of the effect of the ACAT1 S54/69A mutation on PDHA1 acetylation in Cicaprost- and Ang II-stimulated HL-1 cells. **r** Ejection fraction (EF) of aortic constricted-CMIP^−/−^ and LC mice (*n* = 10–14, two-way ANOVA, Tukey multiple comparisons test was used to compare the mean of each group, *P < 0.05). **s** PDH activity in heart tissues from aortic constricted-CMIP^−/−^ mice (*n* = 10, two-way ANOVA, Tukey multiple comparisons test was used to compare the mean of each group, *P < 0.05). **t** Mitochondrial Acetyl-CoA level in heart tissues from aortic constricted-CMIP^−/−^ and LC mice (*n* = 9–10, two-way ANOVA, Tukey multiple comparisons test was used to compare the mean of each group, *P < 0.05). **u** ACAT1 phosphorylation and PDHA1 acetylation levels in heart tissues from aortic constricted-CMIP^−/−^ mice. **v** Ejection fraction (EF) of NS304-treated AAC mice (*n* = 11, two-sided *t* test, *P < 0.05). **w** Effect of NS304 on PDH activity of heart tissues from AAC mice after NS304 treatment (*n* = 6, two-sided *t* test, *P < 0.05). **x** Mitochondrial acetyl-CoA level in heart tissues from NS304-treated AAC mice (*n* = 6 per group, two-sided *t* test, *P < 0.05). **y** ACAT1 phosphorylation and PDHA1 acetylation levels in heart tissues from NS304-treated mice. **z** Mechanistic diagram for IP receptor-mediated glucose oxidation in cardiomyocytes by ACAT1 S54/69 phosphorylation. Data are shown as mean ± SEM

A stable isotope tracer experiment was performed to determine the effect of the IP receptor on glucose/pyruvate oxidation flux in cardiomyocytes. Cay10441 treatment did not significantly alter glucose-6-phosphate (G6P), fructose-6-phosphate (F6P), or lactate production, while it dramatically induced pyruvate accumulation and suppressed generation of TCA cycle intermediates in HL-1 cardiomyocytes (Fig. 1b), indicating that inhibition of IP receptor may impede pyruvate influx into TCA oxidation in cardiomyocytes. Consistently, IP agonist Cicaprost did not alter citrate (Si)-synthase (CS), isocitrate dehydrogenase (IDH), or α-oxoglutarate dehydrogenase complex (α-KGDHC) activities, but markedly increased pyruvate dehydrogenase (PDH) activity in HL-1 cells (Supplementary Fig. S2a–d). Conversely, the IP inhibitor or deficiency suppressed PDH activity in Ang II-treated HL-1 cells and NMCMs (Fig. 1c, Supplementary Fig. S2e). Activation and inhibition of IP did not alter the protein expression of PDH complex enzymatic components (Fig. 1d, Supplementary Fig. S2f). The altering acetylation/deacetylation or phosphorylation/dephosphorylation of PDHA1 regulates PDHC activity.^5^ IP agonist Cicaprost decreased, while antagonist Cay10441 increased serine 293 phosphorylation and acetylation of PDHA1 respectively, without altering acetylation of DLAT and DLD expression (Fig. 1d, e, Supplementary Fig. S2f, g). The acetylation-deficient K → R mutant of PDHA1 (lysine 321 residue^5^), which displays increased PDHC activity, markedly rescued the reduced PDHC activity and mitochondrial acetyl-CoA level in Cay10441-treated HL-1 cells (Fig. 1f, g), implying that IP activation promotes glucose oxidation in cardiomyocytes through inhibiting K321 acetylation of PDHA1.

The protein acetylase GCN5L1 and ACAT1 and the deacetylase Sirt3 directly modulate acetylation and activity of PDHA1. Cicaprost and Cay10441 did not alter ACAT1, GCN5L1 and Sirt3 expression in HL-1 cells (Supplementary Fig. S3a, b). Notably, silencing of ACAT1 attenuated the enhanced PDHA1 acetylation and subsequently restored the reduced PDHC activity in Cay10441-treated HL-1 cells (Fig. 1h, i), while knockdown of GCN5L1 or Sirt3 did not alter IP activity- mediated acetylation/deacetylation of PDHA1 in HL-1 cells (Supplementary Fig. S3c, d), indicating that IP receptor regulates PDH activity through ACAT1-mediated PDHA1 acetylation.

IP receptor is coupled to Gαs, which activates PKA by boosting cellular cAMP.^3^ Indeed, IP activation by Cicaprost increased serine (S) phosphorylation of human ACAT1 in HL-1 cells without markedly affecting its threonine (T) phosphorylation levels, and PKA inhibitor H89 attenuated the Cicaprost-stimulated phosphorylation of ACAT1 and Cicaprost-suppressed acetylation of PDHA1 (Fig. 1j, k). In vitro PKA kinase assay and LC-MS/MS results showed that PKA was able to directly phosphorylate S54 and S69 of human ACAT1 in vitro (Fig. 1l). The non-phosphorylatable S54/69A double mutation restored forskolin-induced ACAT1 phosphorylation, PDHA1 deacetylation and PDH activity in cultured HL-1 (Fig. 1m, n, Supplementary Fig. S4a). The structure of human ACAT1 forms a tetrameric holoenzyme.^6^ The Ser54 side chain is within hydrogen binding distance to the Glu225 side chain near its substrate binding loop. The Ser69 side chain is within hydrogen binding distance to the mainchain carbonyl of Ala107, which is located in a helix close to the substrate binding site of a neighboring ACAT1 subunit (Fig. 1o). Therefore, phosphorylation at Ser54 or Ser69 may disrupt these important interactions and cause defects in substrate binding and enzyme activity. These observations indicate that PKA directly phosphorylates ACAT1 at S54 and S69, thereby inhibiting ACAT1 acetylase activity. Likewise, the non-phosphorylatable S54/69A mutation markedly attenuated these effects of IP agonist Cicaprost on ACAT1 serine phosphorylation, PDHA1 acetylation and PDH complex function in HL-1 cells (Fig. 1p, q, Supplementary Fig. S4b). Therefore, IP receptor regulates PDH activity through PKA-mediated S54/69 phosphorylation of ACAT1 in cardiomyocytes.

Then IP receptor Cardiomyocyte-specific IP knockout (CMIP^−/−^) mice was generated (Supplementary Fig. S5a, b) to explore the effect of IP deletion on pressure overload-induced heart failure. IP deficiency in cardiomyocytes significantly exacerbated AAC-induced cardiac hypertrophy and heart failure in mice (Supplementary Fig. S5c–f, Fig. 1r). We also observed a notable reduction in PDH activity and mitochondrial acetyl-CoA level in heart tissues from aortic-constricted CMIP^−/−^ mice with enhanced PDHA1 acetylation and decreased ACAT1 phosphorylation (Fig. 1s–u). Similarly, IP deficiency in cardiomyocytes significantly exacerbated Ang II-induced cardiac hypertrophy and cardiac dysfunctions in mice (Supplementary Fig. S6a–j).

To test the therapeutic potential of activation of IP receptor on cardiac hypertrophy and heart failure, the clinically available and orally active IP agonist NS-304 was used to treat both AAC and Ang II-infused mice. As anticipated, IP agonist NS304 alleviated AAC and Ang II-induced cardiac hypertrophy and heart failure in mice, by increasing PDH activity through the reduction of ACAT1-dependent PDHA1 acetylation (Supplementary Fig. S7a–d, Fig. 1v–y, Supplementary Fig. S8a–j).

In summary, we had shown the PGI_2_/IP axis improves cardiac glucose oxidation and pressure overload-induced heart failure via PKA/ACAT1/PDHA1 pathway. Our results suggest that IP receptor activation may be an attractive therapeutic strategy for pressure overload-induced heart failure.

### Supplementary information

Supplementary Materials

## Data Availability

All research data of this article are available upon reasonable request by readers.
