# Supplementary material for: I prostanoid receptor activation attenuates pressure overload-induced cardiac hypertrophy by enhancing glucose oxidation
Source: Signal Transduct Target Ther. 2023 Sep 6;8:337. doi: 10.1038/s41392-023-01541-1 (PMC10480462; doi:10.1038/s41392-023-01541-1)
Supplement: Supplementary file 1 — Supplementary Materials [file 41392_2023_1541_MOESM1_ESM.docx]

Supplementary Materials for

I prostanoid receptor activation attenuates pressure overload-induced cardiac hypertrophy by enhancing glucose oxidation

Qian Liu^#^, Guizhu Liu^#^, Yujuan Zhuo, Shihong Chen, Yinghong Zheng, Kai Zhang, Song Xiang, Jiangping Song, Liming Yang*, Ying Yu*.

Correspondence to: [yuying@tmu.edu.cn](mailto:yuying@tmu.edu.cn) and limingyanghmu@163.com

**This PDF file includes:**

Materials and Methods

Figures. S1 to S8

Tables S1 to S2

**Materials and Methods**

**Animals**

All animals were of the C57/BL6 background and approved by the Institutional Animal Care and Use Committee of the Tianjin Medical University (NO. TMUaMEC 2020020) and performed according to the guidelines from the National Institutes of Health (NIH) Guide for the Care and Use of Laboratory Animals. All mice were housed five per cage in controlled environment with a constant temperature of 25°C and a humidity of 55%–60% on a 12:12-h light/dark cycle. To generate a tissue-specific IP knockout mouse, two loxP sites were inserted flanking exon 1 and leading to a frameshift causing loss of function of the mouse IP gene after Cre recombination (Supplementary Fig.S 5a-c). Cas9 and specific gRNA were co-injected into fertilized eggs with donor vector to create the floxed (fl) IP allele. To selectively delete IP in cardiomyocytes, IP^fl/fl^ mice were crossed with α-MHC-Cre mice^1^.

IP^fl/fl^ mice were identified using primers: 5’-GGC TAT CCA TCG CC AAA GTG TTA G-3’, 5’-CTG GTC CCT GGA CAC AAT TCA CTC-3’ and 5’-ATA GTG GGA GGG GAC AGC ACA AG-3’. PCR reaction: 94°C 3min, 33 cycles of (94°C 30sec, 60°C 35sec, 72 °C 35sec), 72 °C 5min, hold at 4°C. α-MHC-Cre mice were identified using primers: 5’-TGG TGG TGT AGG AAA GTC AGG-3’, 5’-ACA TGT CCA TCA GGT TCT TG-3’, 5’-AAG TGA GTT TGC ATG GCG CAG C-3’and 5’-CCC TTT AGC CCC TTC CCT CTG-3’. PCR reaction: 94°C 3min, 35 cycles of (94°C 30sec, 60°C 30sec, 72 °C 30sec), 72 °C 2min, hold at 4°C.

**Mouse abdominal aortic constriction (AAC)**

Abdominal aortic constriction (AAC) was performed according to a previously reported protocol^2,3^. Briefly, male mice (7–8 weeks old) were anesthetized with 200 mg/kg Avertin by intraperitoneal injection. Aortic constriction was performed by ligation of the abdominal aorta above both renal arteries with a 27-gauge needle using silk sutures (6–0). The needle was then removed immediately. For the sham operation, silk sutures were passed under the aorta without ligation. Cardiomyocyte-specific IP-knockout mice (CMIP^-/-^) were compared with littermate controls (LC, IP^fl/fl^ mice). The IP agonist selexipag (NS304, 3 mg/kg) was administered to mice by gavage twice per day till sacrifice as previously described^4^.

**Ang II infusion in mice**

Male mice (8-10 weeks) received Ang II (1 μg/kg/min, Sigma Aldrich) or saline (control) continuously via Alzet mini-osmotic pumps (model 2004; Durect Corporation) for up to 28 days. Pumps were prepared and subcutaneously implanted into the mice following the manufacturer’s instructions.

**Echocardiography**

Transthoracic 2-dimensional (2D) echocardiography was performed 4 weeks after surgery using an echocardiograph (Vevo2100) equipped with an MS400 linear array transducer. Mice were anesthetized by isoflurane inhalation. Body temperature was maintained at 37ºC. Two-dimensional parasternal long axis views of the left ventricle were obtained for guided M-mode measurements of various parameters. Based on these data, the LV ejection fraction (EF) and fractional shortening (FS) were calculated. Once the measurements were completed, heart tissues were excised, weighed, and washed with ice-cold PBS. The hearts were then either snap-frozen in liquid nitrogen for further biochemical analysis or fixed for histological staining.

**Stable isotope labeling and metabolite extraction**

[U-^13^C]-glucose was used for energy metabolism analysis. HL-1 cells were seeded into 10 cm plates and pretreated with 2 μM Cay10441 for 12 h. Then, the culture medium was changed to fresh DMEM (Thermo Fisher Scientific, Cat No.11966-025) containing 2 mg/mL [U-^13^C]-glucose (Cambridge Isotope Laboratories, Cat NO. CLM-1396). Cells were then stimulated with 1 μM Ang Ⅱ (angiotensin Ⅱ) and Cay10441. 24 h later, medium was aspirated, and HL-1 cells were washed with cold PBS three times and quenched with 1.5 mL of pre-cooled extraction solution (methanol: acetonitrile: water, v/v/v=2:2:1). Cell supernatants were collected by centrifugation for [U-^13^C]-glucose metabolite analysis by ultra‐high performance liquid chromatography‐high resolution mass spectrometry (UPLC-HRMS). LC-MS acquisition, raw data analysis, and normalization were performed as previously described^5^. Integrated peak areas for each metabolite were used to calculate relative abundance ratios. For individual metabolites, the peak intensities of each ^13^C isotopologue were used to examine the ^13^C isotopologue distributions.

**Histological analysis**

Heart tissues were fixed in a 4% paraformaldehyde solution for 2 days. Hematoxylin and eosin (H&E) and wheat germ agglutinin (WGA) staining (5 μm paraffin sections) were used to examine histopathology. For WGA analysis, deparaffinized slices were rehydrated, then blocked with 5% normal goat serum and 1% BSA in PBS for 1 h. Cell membranes were stained with 5 μg/mL Alexa Fluor 488-conjugated WGA (Thermo Fisher Scientific, Cat No. W11261) for 1 h and washed three times in PBST. Images were captured with Zeiss Confocal Laser Scanning Microscope. Cell cross area were measured using Image J 6.0.

**Cell culture and drug treatment**

HL-1 mouse atrial myocytes were purchased from Sigma-Aldrich (SCC065, Merck, Milan, Italy). Cells were cultured in a humidified atmosphere of 5% CO_2_ at 37°C in Claycomb medium (Sigma Aldrich), as described previously ^6^. The drugs used to stimulate the cells were as follows: 2 μM BWA868C (DP1 antagonist); 2 μM CAY10595 (DP2 antagonist); 2 μM SC51322 (EP1 antagonist); 2 μM PF04418948 (EP2 antagonist); 2 μM L798,106 (EP3 antagonist); 2 μM L-161,982 (EP4 antagonist), 2 μM AL-8810 (FP antagonist); 2 μM CAY10441 (IP antagonist); 2 μM U46619 (TP antagonist); 1 or 10 μM Cicaprost (IP agonist); 10 μM Forskolin (adenylate cyclase activator); and 10 μM H89 (PKA inhibitor).

**Primary cardiomyocyte and myofibroblast isolation, culture, and treatment**

Neonatal rat ventricular myocytes (NRVMs) isolation and neonatal mouse cardiomyocytes (NMCMs) from were isolated from ventricles of 1 to 2 days old Sprague-Dawley rats and mouse were isolated and cultured as previously reported^7^. Cardiomyocyte hypertrophy was induced by 1 μM Ang Ⅱ for 48h.

**Mitochondria Isolation**

Crude mitochondria from hearts tissues and HL-1cells were prepared by differential centrifugation according to a manufacturer’s protocol (Beyotime), as described previously^8^. Mitochondrial protein concentrations were determined with the BCA Protein Assay Kit (Thermo Fisher Scientific).

**Acetyl-Coenzyme A measurement**

Acetyl-Coenzyme A product in mitochondria was determined using an Solarbio Kit according to the manufacturer’s instructions, as described previously^9^. Briefly, isolated mitochondria were collected and incubated in extraction buffer for 30 min. The mitochondria were subjected to sonication and centrifuged at 8000× g at 4 °C for 10 min. The supernatants were collected and supplemented with acetyl-CoA assay buffer. The 340 nm absorbance values were measured at 20 s (OD20s) and 80 s (OD80s). The difference between OD80s and OD20s was used to calculate the relative level of acetyl-CoA. The Acetyl-Coenzyme A production were normalized by total mitochondria protein.

**Measurement of cellular PDH activity**

PDH activity was assessed with commercial kits (Solarbio, Beijing, China) as previously described^10^. HL-1 cells were seeded into 6-cm dishes and transfected with or without plasmids for 36 h prior to co-treatment with different drugs for 12 h. *In vivo*, heart tissues from AAC mice were homogenized in cold PBS, and PDH activity was determined as described above. The data were normalized by protein concentration, cell numbers, or tissue mass.

**Seahorse XF-24 metabolic flux analysis**

HL-1 cardiomyocytes, NRVMs or NMCMs were seeded into Seahorse XF-24 plates at a density of 50,000 cells per well. Cells were treated with PG receptor antagonist or IP agonist for 24 h prior to the assay. The mitochondrial oxygen consumption rate (OCR) and extracellular acidification rate (ECAR) were determined through XF analysis, as described previously^11,12^. Before initiating the assay, cells were washed and incubated at 37℃ without CO_2_ for one hour to allow equilibration. The extracellular acidification rate (ECAR) and oxygen consumption rate (OCR) in real-time were measured using the glycolysis stress test kit and the Mito stress test kit, according to the manufacturer’s instructions. For OCR measurement, Oligomycin (1 μM), FCCP (1 μM), and Antimycin A (10 μM) plus rotenone (1 μM) were used. For ECAR measurement, glucose (10 mM), oligomycin (1 μM), and 2-deoxyglucose (50 mM) were used.

***In vitro* kinase assay and LC-MS/MS acquisition**

For the *in vitro* PKA phosphorylation assay, reactions were carried out with 1.4 μl ATP (10 mM), 0.2 μl 2,500,000 units/ml cAMP-dependent protein kinase (PKA), catalytic subunit, 2 μl 10×NEB Buffer (New England Biolabs), and 15 μg synthetic peptide at 30°C for 30 minutes. Then, the samples were analyzed by LC-MS/MS for determining phosphorylation sites of synthetic peptides. The following synthetic peptides were generated by Genscript Biochem (Nanjing, China): 54WT, ATRTPIGSFLGSLSLLPATK; S54A, ATRTPIGAFLGSLSLLPATK; 69WT, SLLPTAKLGSIAIQGAIEKA; S69A SLLPTAKLGAIAIQGAIEKA.

**RNA interference and gene overexpression**

Cells were transiently transfected with 200 pmol ACAT1, GCN5L1, Sirt3, or negative control siRNA per well in a 6 cm dish using EL Transfection Reagent (Transgen) according to the manufacturer’s instructions. And siRNA-specific sequences for ACAT1 and Sirt3 mRNA were designed by Shanghai GenePharma Co., Ltd. In addition, cells were seeded into a 10 cm dish and transfected with 24 μg of human ACAT1 or PDHA1 expression plasmids per well using EL Transfection Reagent (Transgen). The most effective siRNA sequences of ACAT1 and Sirt3 are listed in Supplementary Table 1.

**Immunoprecipitation**

Cells or heart tissues were lysed for 30 min with ice-cold Western/immunoprecipitation Lysis buffer (Beyotime) supplemented with protease inhibitor cocktail. Whole cell lysates were then centrifuged at 14,000 × g for 15 min at 4°C. The soluble fraction was collected, and its protein concentration determined by BCA assay. For immunoprecipitation, 1 mg protein in lysis buffer was immunoprecipitated overnight with anti-FLAG magnetic beads (Bimake, Cat No. B26101) or anti-HA magnetic beads (Bimake, Cat No. B26301) at 4°C. For untagged protein, the protein supernatant was incubated with primary antibodies or IgG antibody and protein A/G-magnetic beads (Bimake, Cat No. B23201), rotating at 4°C overnight. Next, the beads were washed three times with TBS and denatured by 50 uL SDS loading buffer. Immunoprecipitated complexes were subjected to Western blot for detection of target proteins.

**Western blotting**

Proteins from total cell and tissue lysates were separated by 10% SDS-PAGE and transferred electrophoretically onto Immobilon-P polyvinylidene difluoride (PVDF) membranes (Millipore). The membranes were probed with rabbit anti-ACAT1 polyclonal antibody (Proteintech, Cat No.16215-1-AP.), rabbit anti-PDHA1 monoclonal antibody (ABclonal, Cat No. A13687), rabbit anti-PDHA1 (phospho S293) polyclonal antibody (Abcam, Cat No. ab92696), rabbit anti-DLAT polyclonal antibody (Proteintech, Cat No. 13426-1-AP), rabbit anti-DLD polyclonal antibody (Proteintech, Cat No. 16431-1-AP), rabbit anti-HA monoclonal antibody (Cell Signaling Technology, Cat No. 3724), rabbit anti-DDDDK monoclonal antibody (Cell Signaling Technology, Cat No. 14793), rabbit anti-acetylated-lysine monoclonal antibody (Cell Signaling Technology, Cat No. 14793), rabbit anti-phosphoserine polyclonal antibody (Abcam, Cat No. ab9332), mouse anti-phosphothreonine monoclonal antibody (Cell Signaling Technology, Cat No. 9386), rabbit anti-phospho-(Ser/Thr) polyclonal antibody (Abcam, Cat No. ab17464), followed by incubation with either anti-rabbit or anti-mouse IgG secondary antibodies conjugated to horseradish peroxidase (Protein Tech Group, Inc.) and detection using the ECL system (Pierce).

**RNA extraction and quantitative real‐time polymerase chain reaction (qRT‐PCR)**

Total RNA was isolated from cells using TRIzol reagent (Invitrogen) according to the manufacturer's instructions. RNA concentration and purity were assessed using a NanoDrop 2000 (Thermo Fisher). Total RNA (2.0 μg) was reverse transcribed with reverse transcription reagent kits (Takara) and oligo(dT)18 primers (Takara) as recommended. qRT-PCR was performed using a LightCycle@ 480 system (Roche) and Hieff Unicon® TaqMan multiplex qPCR master mix (Yeasen) as described by the manufacturer. Raw data were normalized to peptidylprolyl isomerase A (PPIA) and presented as relative expression levels calculated by the 2^-ΔΔ^Ct method. All primers for qRT-PCR are shown in Supplementary Table 2.

**Statistical analysis**

All data were analyzed by GraphPad Prism 9.0 software and expressed as means ± SEM. Data distribution was evaluated by Shapiro-Wilk normality test. P>0.05 indicates that variables follow a normal distribution in the population. Normally distributed data were analyzed with the unpaired, 2-tailed t test (two groups), 1-way (more than two groups and only one independent variable) or 2-way ANOVA (more than one independent variable) followed by Tukey, Dunnett, or Sidak multiple comparisons test. For data not following a normal distribution, the unpaired 2-tailed Mann-Whitney U test (two groups) or Kruskal-Wallis test (three or more groups) was used, followed by Dunn *post hoc* test.

**References**

1 Agah, R. *et al.* Gene recombination in postmitotic cells. Targeted expression of Cre recombinase provokes cardiac-restricted, site-specific rearrangement in adult ventricular muscle in vivo. *J Clin Invest* **100**, 169-179 (1997).

2 van der Pol, A., van Gilst, W. H., Voors, A. A. & van der Meer, P. Treating oxidative stress in heart failure: past, present and future. *Eur J Heart Fail* **21**, 425-435 (2019).

3 Zhang, M. *et al.* Both cardiomyocyte and endothelial cell Nox4 mediate protection against hemodynamic overload-induced remodelling. *Cardiovasc Res* **114**, 401-408 (2018).

4 Batchu, S. N. *et al.* Prostaglandin I2 Receptor Agonism Preserves beta-Cell Function and Attenuates Albuminuria Through Nephrin-Dependent Mechanisms. *Diabetes* **65**, 1398-1409 (2016).

5 Liu, X., Romero, I. L., Litchfield, L. M., Lengyel, E. & Locasale, J. W. Metformin Targets Central Carbon Metabolism and Reveals Mitochondrial Requirements in Human Cancers. *Cell Metab* **24**, 728-739 (2016).

6 Lee, T. W., Lee, T. I., Lin, Y. K., Kao, Y. H. & Chen, Y. J. Calcitriol downregulates fibroblast growth factor receptor 1 through histone deacetylase activation in HL-1 atrial myocytes. *J Biomed Sci* **25**, 42 (2018).

7 Wang, X. *et al.* PCSK9 regulates pyroptosis via mtDNA damage in chronic myocardial ischemia. *Basic Res Cardiol* **115**, 66 (2020).

8 Cui, W. *et al.* 20-HETE synthesis inhibition attenuates traumatic brain injury-induced mitochondrial dysfunction and neuronal apoptosis via the SIRT1/PGC-1alpha pathway: A translational study. *Cell Prolif* **54**, e12964 (2021).

9 Zhou, W. J. *et al.* TIGAR promotes neural stem cell differentiation through acetyl-CoA-mediated histone acetylation. *Cell Death & Disease* **10 (**2019).

10 Guitart, M. *et al.* FATP1 localizes to mitochondria and enhances pyruvate dehydrogenase activity in skeletal myotubes. *Mitochondrion* **9**, 266-272 (2009).

11 Liu, Y., Nguyen, P., Baris, T. Z. & Poirier, M. C. Molecular analysis of mitochondrial compromise in rodent cardiomyocytes exposed long term to nucleoside reverse transcriptase inhibitors (NRTIs). *Cardiovasc Toxicol* **12**, 123-134 (2012).

12 Wang, L. X. *et al.* Glucose transporter 1 critically controls microglial activation through facilitating glycolysis. *Mol Neurodegener* **14 (**2019).


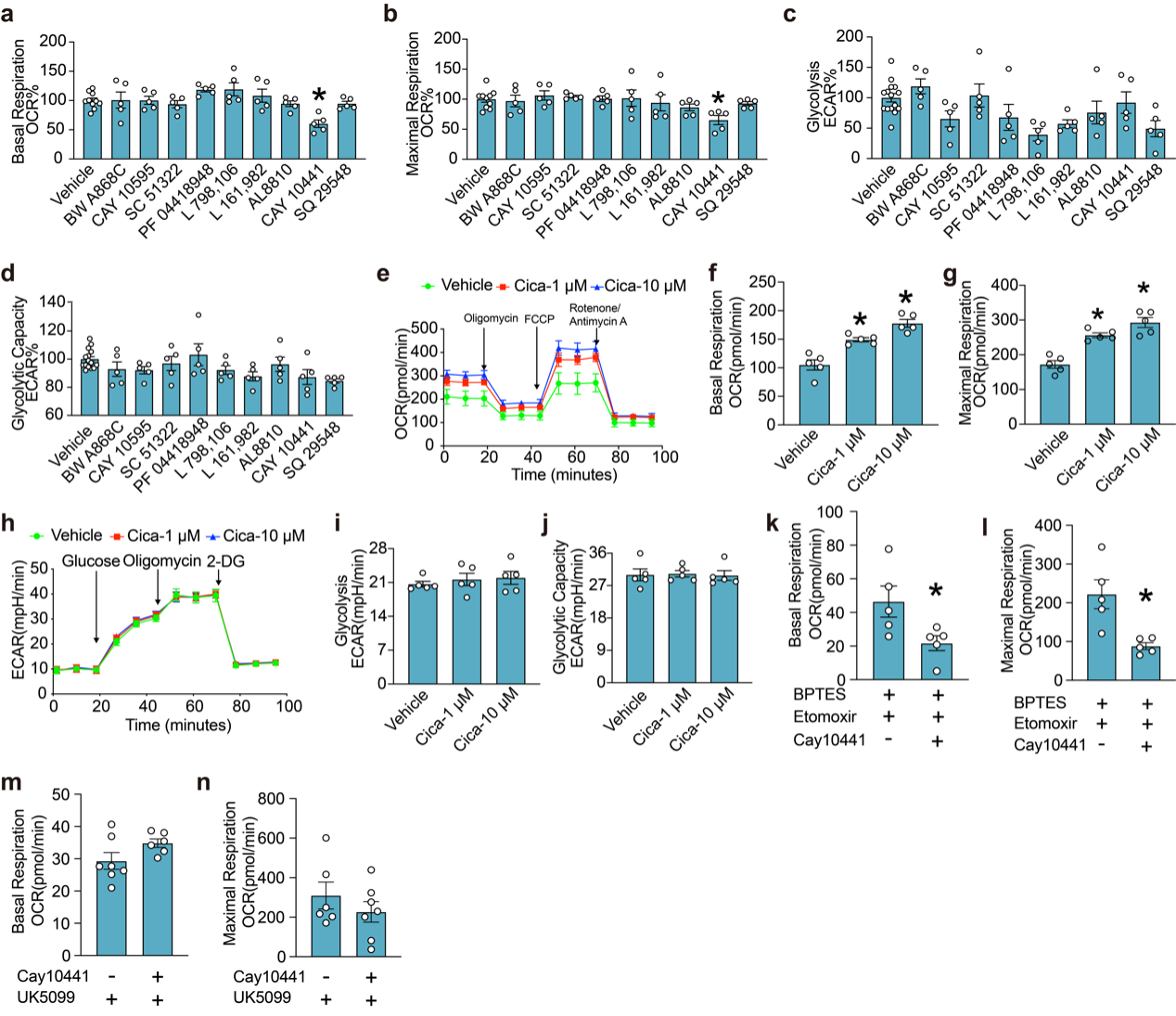


**Supplementary Fig.1**

**IP receptor promotes glucose aerobic oxidation in cardiomyocytes.**

**a-b** Effect of different prostanoid receptor antagonists on basal and maximal OCR of NRVMs. NRVMs were treated with 2 μM PG receptor antagonists (DP1 inhibitor, BWA868C; DP2 inhibitor, CAY10595; EP1 inhibitor, SC-51322; EP2 inhibitor, PF-04418948; EP3 inhibitor, L-798,106; EP4 inhibitor, L-161,982; FP, AL8810; IP inhibitor, CAY10441; TP inhibitor, SQ29548) combined with 1 μM Ang II for 24 h. OCR was monitored in real time under basal conditions, following inhibition of ATP synthase (with 1 μM oligomycin) and uncoupling of the electron transport chain with 0.5 μM FCCP and complexes I/III with 2 μM rotenone/antimycin A. The experiment was performed in the presence of glucose, pyruvate and glutamine. (n≥5, 1-way ANOVA, Holm Sidak multiple comparisons test, *P<0.05). **c-d** Effect of prostanoid receptor antagonists on glycolysis and glycolytic capacity in NRVMs. NRVMs were treated with 2 μM PG receptor antagonists and with 1 μM Ang II for 24 h. Basal ECAR measurement was measured in XF assay medium with pyruvate and glutamine, but without glucose, followed by the addition of glucose (10 mM), oligomycin (1 μM), and 2-DG (50 mM) for the indicated times to determine different parameters of glycolysis functions (n≥5, 1-way ANOVA, Dunn’s multiple comparisons test, *P<0.05). **e-g** Basal OCR and maximum respiration in Cicaprost treated-HL-1 cells. The experiment was performed in the presence of glucose, pyruvate and glutamine. (n=5, 1-way ANOVA, Tukey multiple comparisons test was used to compare the mean of each group, *P<0.05). **h-j** ECAR and glycolytic capacity in Cicaprost-treated HL-1 cells (n=5, 1-way ANOVA was used to compare the mean of each group, *P<0.05). **k-l** FA oxidation and glutamine import were blocked by pretreatment with Etomoxir and BPTES, and basal respiration and maximum respiration were measured in Cay10441-treated NRCMs. (n=5, two-sided t test, *P<0.05). The experiment was performed in the presence of glucose, pyruvate, glutamine, and palmitate-BSA. **m-n** Effect of Cay10441 on basal OCR and maximum respiration in mitochondrial pyruvate carrier inhibitor UK5099-treated NRCMs. The experiment was performed in the presence of glucose, pyruvate, glutamine, and palmitate-BSA. (n=6-7, two-sided Mann-Whitney U test, *P<0.05). All values are represented as mean ± SEM.


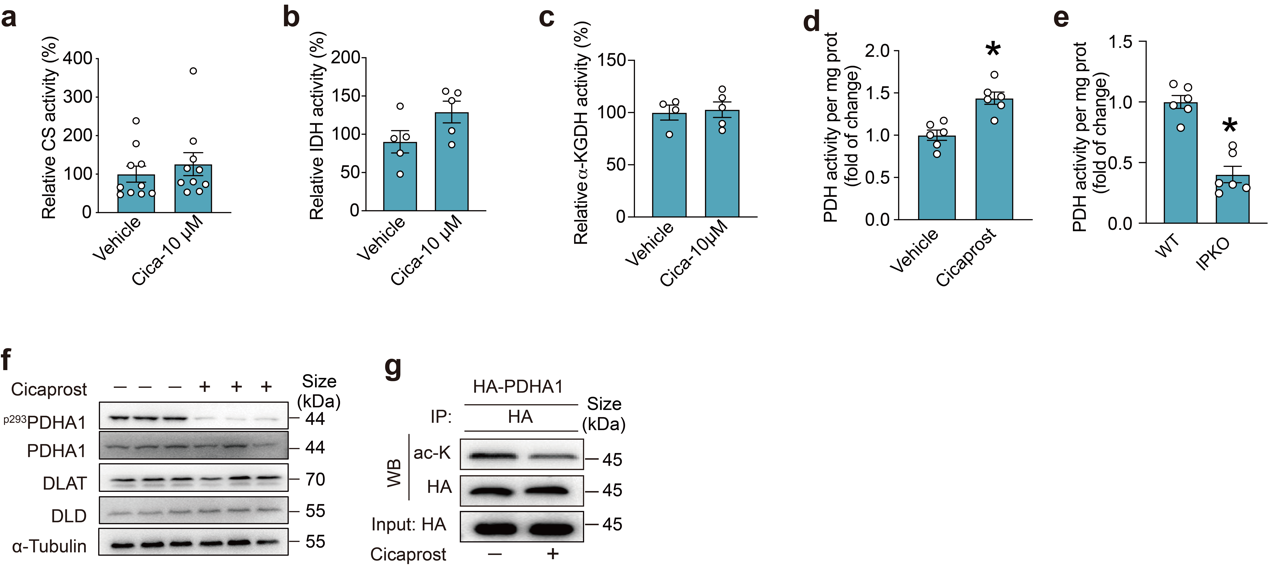


**Supplementary Fig.2**

**IP receptor facilitates glucose oxidation in cardiomyocytes via enhancing PDH activity through inhibiting PDHA1 acetylation.**

**a-c** Activities analyses of Citrate (Si)-synthase (CS, **a**), isocitrate dehydrogenase (IDH, **b**) and α-oxoglutarate dehydrogenase complex (α-KGDH, **c**) activities in Cicaprost(Cica)- and Ang II-stimulated HL-1 cells. (n=4-5, two-sided t test, *P<0.05). **d** Effect of IP agonist Cicaprost on pyruvate dehydrogenase (PDH) activity in Ang II-stimulated HL-1 cells (n=6, two-sided t test, *P<0.05). **e** Effect of IP deficiency on PDH activity in neonatal mouse cardiomyocytes with Ang II treatment (n=6, two-sided Mann-Whitney U test, *P<0.05). **f** Western blot analyses of the effect of Cicaprost on protein expression levels of PDHA1, PDHA1-p293S, DLAT, and DLD in Ang II-stimulated HL-1 cells. **g** Western blot analyses of effect of Cicaprost on PDHA1 acetylation in Ang II-stimulated HL-1 cells. All values are represented as mean ± SEM.

**
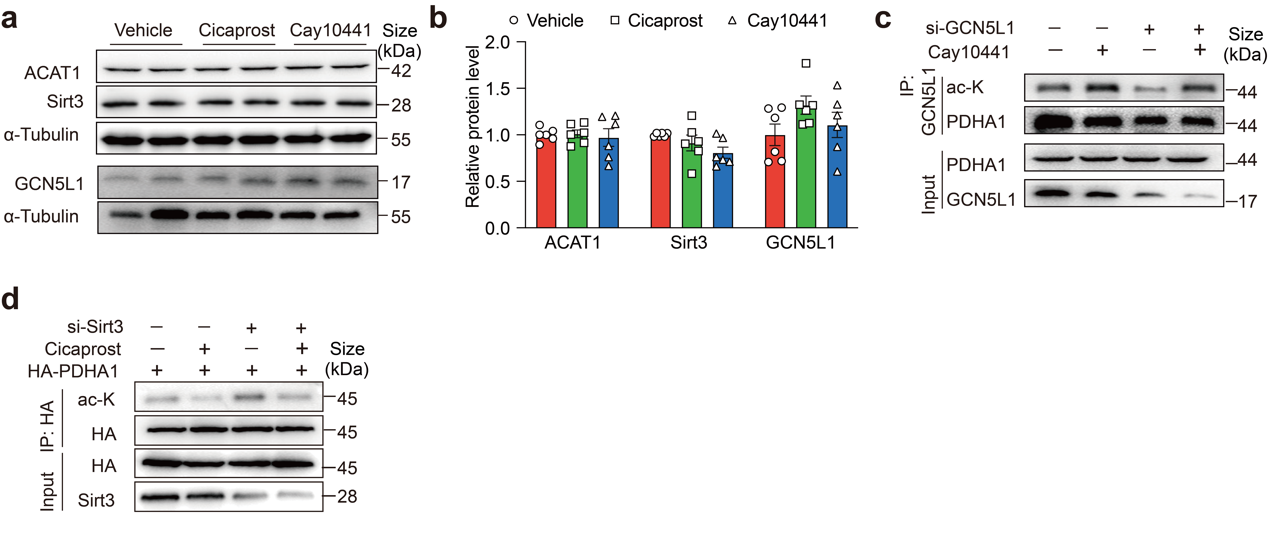
**

**Supplementary Fig. 3**

**IP receptor suppresses PDHA1 acylation in Ang** **II-stimulated cardiomyocytes through the acetyltransferase ACAT1.**

**a** Western blot analysis of the effect of Cicaprost or Cay10441 treatment on protein expression of ACAT1, GCN5L1 and Sirt3 in Ang II-stimulated HL-1 cells. **b** The relative protein density levels were quantified by ImageJ software as shown in **a** (n=6, two-sided t test, *P<0.05). **c** Effect of GCN5L1 silencing on PDHA1 acetylation in Cay10441-treated HL-1 cells. **d** Western blot analysis of the effect of Sirt3 silencing on PDHA1 acetylation in Cicaprost-stimulated HL-1 cells. All values are represented as mean ± SEM.


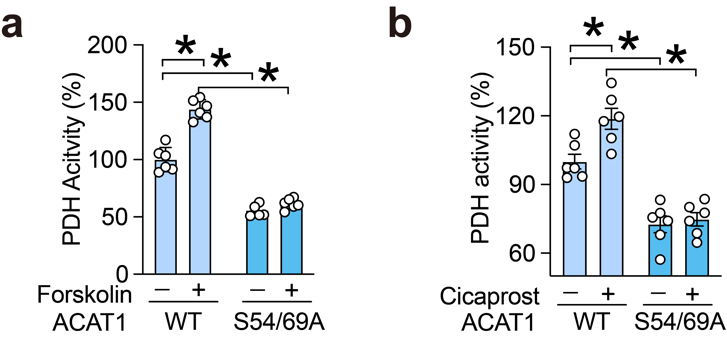


**Supplementary Fig. 4**

**Forskolin promotes PDH activity in cardiomyocytes through S54/69 phosphorylation of ACAT1.**

**a** Effect of the ACAT1 S54/S69A double mutation on forskolin-induced PDH activity in Ang II-stimulated HL-1 cells (n=6, 2-way ANOVA, Tukey multiple comparisons test was used to compare the mean of each group, *P<0.05). **b** Effect of the ACAT1 S54/69A mutation on PDH activity in Cicaprost- and Ang II-stimulated HL-1 cells (n=6, 2-way ANOVA, Tukey multiple comparisons test was used to compare the mean of each group, *P<0.05). All values are represented as mean ± SEM.

**
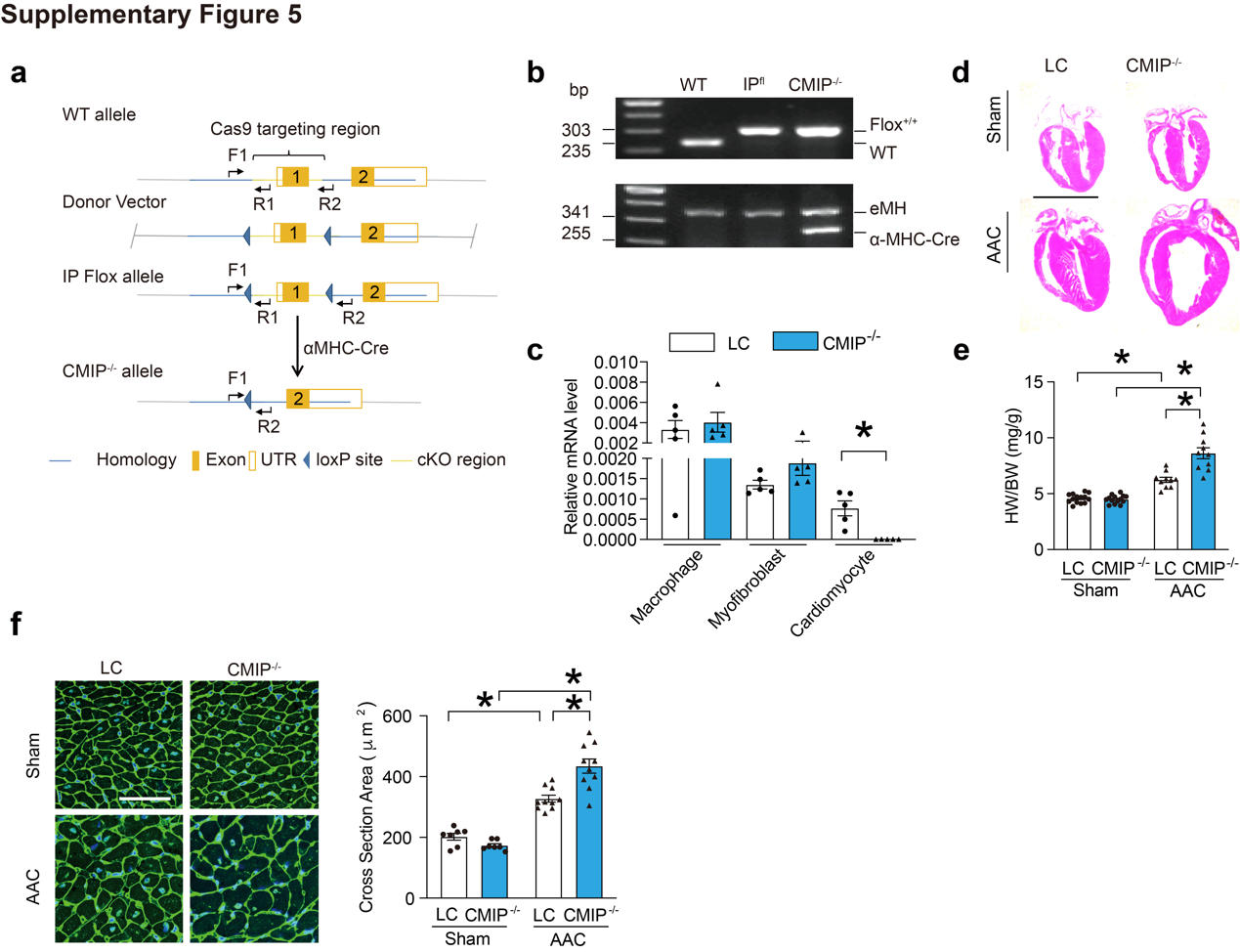
**

**Supplementary Fig. 5**

**Cardiac IP deficiency accelerates AAC-induced hypertrophy and impairs heart function in mice via suppression of PDH activity.**

**a** Schematic diagram of construction strategy used to generate cardiomyocyte-specific IP-deficient (CMIP^-/-^) mice. **b** PCR analysis on genomic DNA derived from CMIP^-/-^ mice and littermate controls (LC). **c** IP mRNA levels in different type cells from CMIP^-/-^ and LC mice. (n=5, two-sided t test, *P<0.05). **d** Representative hematoxylin and eosin (H&E) staining images of heart sections from aortic constricted-CMIP^-/-^ mice. LC, littermate controls. Scale bar: 5 mm. **e** The heart weight /body weight (HW/BW, mg/g) ratio of aortic constricted-CMIP^-/-^ mice (n=10-14, 2-way ANOVA, Tukey multiple comparisons test was used to compare the mean of each group, *P<0.05). **f** Representative WGA staining images (left) and quantification of relative cardiomyocyte sizes of aortic constricted-CMIP^-/-^ mice (right). Scale bar: 50 μm (n=7 -10, 2-way ANOVA, Tukey multiple comparisons test was used to compare the mean of each group, *P<0.05). All values are represented as mean ± SEM.

**
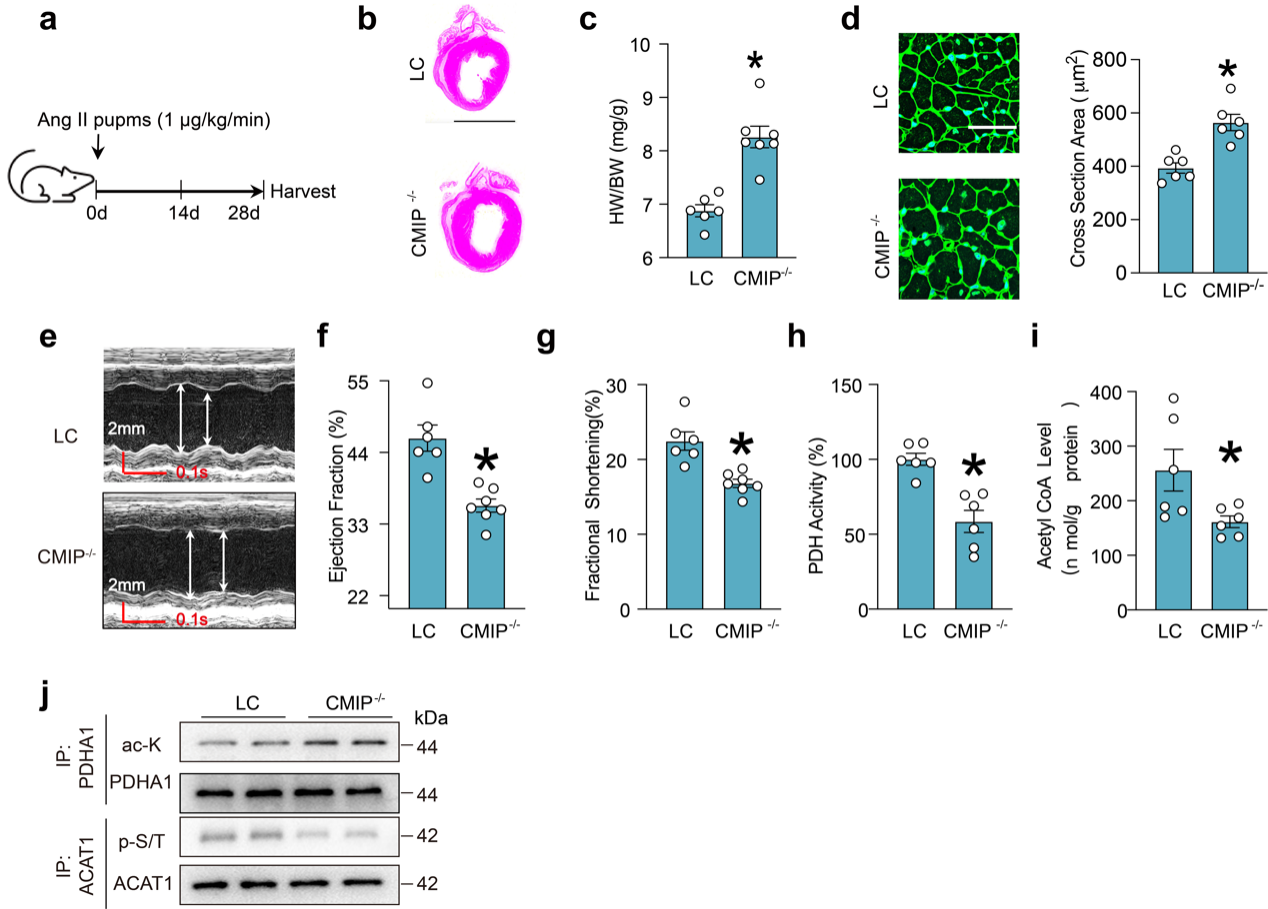
**

**Supplementary Fig. 6**

**Cardiac IP deficiency accelerates Ang II-induced hypertrophy and impairs heart function via in mice suppression of PDH activity.**

**a** Schematic diagram of Ang II fusion in mice. **b** Representative H&E staining images of heart sections from littermate control (LC) and CMIP^-/-^ mice after Ang II infusion. Scale bar: 5 mm. **c** The heart weight/body weight (HW/BW, mg/g) ratio of littermate controls (LC) and CMIP^-/-^ mice after Ang II infusion (n=6-7, two-sided t test, *P<0.05). **d** Representative WGA staining images (left) and quantification of relative cardiomyocyte sizes of LC and CMIP^-/-^ mice after Ang II infusion. Scale bar: 50 μm (n=6-7, two-sided t test, *P<0.05). **e-g** Representative images showing M mode echocardiography of LC and CMIP^-/-^ mice after Ang II infusion, Ejection fraction (EF) and Fractional shortening (FS) at day 28. Scale bars indicate 2 mm and 0.1 s. (n=6-7, two-sided t test, *P<0.05). **h** PDH activity in heart from LC and CMIP^-/-^ mice after Ang II infusion. (n=6, two-sided t test, *P<0.05). **i** Mitochondrial Acetyl-CoA level in heart tissues from LC and CMIP^-/-^ mice after Ang II infusion. (n = 6, two-sided t test, *P<0.05). **j** Phosphorylated ACAT1 and acetylated PDHA1 levels in heart tissues from LC and CMIP^-/-^ mice after Ang II infusion. All values are represented as mean ± SEM.


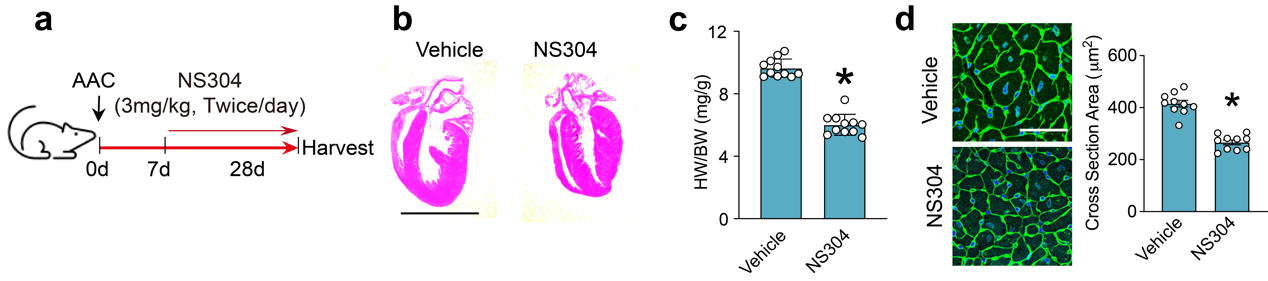
**Supplementary Fig.7**

**IP agonist NS304 alleviates AAC-induced cardiac failure in mice.**

**a** Schematic diagram of NS304 administration in AAC mice. 1 week after AAC surgery, NS304 was administrated to the mice twice per day for 21 days. **b** Representative H&E staining images of heart sections from NS304-treated AAC mice. Scale bars: 5 mm. **c** Effect of NS304 treatment on HW/BW ratio of AAC mice (n=11, two-sided t test, *P<0.05). **d** Representative WGA staining (green, left) and quantification of cardiomyocyte size (right) in heart tissues from NS304-treated AAC mice. Scale bars: 50 μm (n=10, two-sided t test, *P<0.05). All values are represented as mean ± SEM.


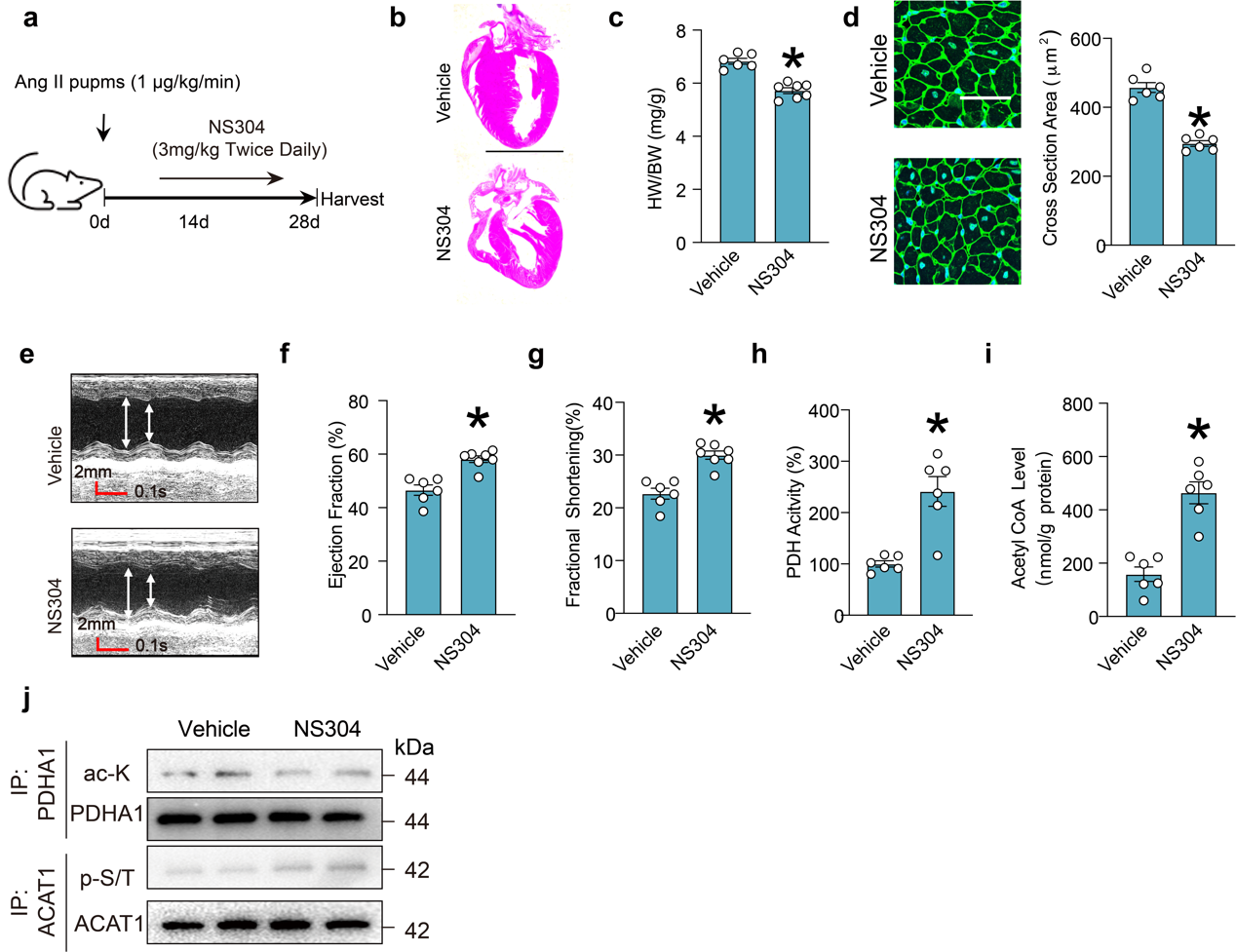


**Supplementary Fig. 8**

**IP agonist NS304 alleviates Ang II-induced cardiac hypertrophy in mice. a** Schematic diagram of NS304 administration in Ang II-infused mice. NS304 was administrated to the mice twice a day for 28 days. **b** Representative H&E staining images of hearts from NS304-treated mice after Ang II infusion. Scale bars: 5 mm. **c** Effect of NS304 treatment on HW/BW ratio of mice. (n=6-7, two-sided t test, *P<0.05). **d** Representative WGA staining (left) and quantification of cardiomyocyte size (right) in heart tissues. Scale bars: 50 μm. (n=6, two-sided t test, *P<0.05). **e** Representative images showing M mode echocardiography of NS304-treated mice with Ang II infusion. Scale bars indicate 2 mm and 0.1 s. **f-g** Ejection fraction (EF) and Fractional shortening (FS) of NS304-treated mice. (n=6-7, two-sided t test, *P<0.05 compared to the Vehicle group). **h** Effect of NS304 on PDH activity of heart tissues from Ang II-infused mice. (n=6, two-sided t test, *P<0.05). **i** Effect of NS304 treatment on mitochondrial acetyl-CoA level in hearts tissues from Ang II-infused mice. (n=6, two-sided t test, *P<0.05). **j** Western blot an analysis of the effect of NS304 treatment on ACAT1 phosphorylation and PDHA1 acetylation in heart tissues from Ang II-infused mice. All values are represented as mean ± SEM.

**Supplementary Table 1.** Nucleotide sequences of siRNA duplexes used for transfection of HL-1 cells.

| **Duplex no.** | **Forward sequences** | **Reverse sequences** |
| --- | --- | --- |
| Sirt3 Duplex 1 | CCAUCUUUGAACUAGGCUUTT | AAGCCUAGUUCAAAGAUGGTT |
| Sirt3 Duplex 2 | ACUCCCAUUCUUCUUUCACTT | GUGAAAGAAGAAUGGGAGUTT |
| ACAT1 Duplex 1 | CAUGGGUAAUGUUCUACAATT | UUGUAGAACAUUACCCAUGTT |
| ACAT1 Duplex 2 | GUGGACAUCAGGAUGUGAUTT | AUCACAUCCUGAUGUCCACTT |
| ACAT1 Duplex 3 | CAAUGCCAGUACACUGAAUTT | AUUCAGUGUACUGGCAUUGTT |

**Supplementary Table 2.** Mouse oligonucleotide primers for real-time PCR.

| **Gene** | **Speices** | **Forward primer** | **Reverse primer** |
| --- | --- | --- | --- |
| IP | Mouse | CGGGCACGAGAGGATGAAGTTTA | GGTTGAAGGCGTTGAAGCGGAAGG |
| PPIA | Mouse | GAGCTGTTTGCAGACAAAGTTC | CCCTGGCACATGAATCCTGG |
